# A Japanese case of oculopharyngeal muscular dystrophy (OPMD) with PABPN1 c.35G > C; p.Gly12Ala point mutation

**DOI:** 10.1186/s12883-021-02300-x

**Published:** 2021-07-05

**Authors:** Yo-suke Nishii, Yu-ichi Noto, Rei Yasuda, Takamasa Kitaoji, Shinji Ashida, Eijirou Tanaka, Narihiro Minami, Ichizo Nishino, Toshiki Mizuno

**Affiliations:** 1 Department of Neurology, Graduate School of Medical Science, Kyoto Prefectural University of Medicine, 465 Kajii-cho, Kamigyo-ku, Kyoto, 602-0841 Japan; 2 Department of Neuromuscular Research, National Institute of Neuroscience, National Center of Neurology and Psychiatry, 4-1-1 Ogawa-Higashi, Kodaira, Tokyo 187-8502 Japan

**Keywords:** Oculopharyngeal muscular dystrophy (OPMD), Poly(a) binding protein nuclear 1 (PABPN1), Point mutation, Muscle ultrasound, Asian case

## Abstract

**Background:**

Oculopharyngeal muscular dystrophy (OPMD) is a late-onset muscular dystrophy characterised by slowly progressive ptosis, dysphagia, and proximal limb muscle weakness. A common cause of OPMD is the short expansion of a GCG or GCA trinucleotide repeat in *PABPN1* gene.

**Case presentation:**

A 78-year-old woman presented with ptosis and gradually progressive dysphagia. Her son had the same symptoms. A physical examination and muscle imaging (MRI and ultrasound) showed impairment of the tongue, proximal muscles of the upper limbs, and flexor muscles of the lower limbs. Needle-electromyography (EMG) of bulbar and facial muscles revealed a myopathic pattern. Based on the characteristic muscle involvement pattern and needle-EMG findings, we suspected that the patient had OPMD. Gene analysis revealed *PABPN1* c.35G > C point mutation, which mimicked the effect of a common causative repeat expansion mutation of OPMD.

**Conclusion:**

We herein describe the first reported Japanese case of OPMD with *PABPN1* point mutation, suggesting that this mutation is causative in Asians as well as in Europeans, in whom it was originally reported.

## Background

Oculopharyngeal muscular dystrophy (OPMD) is a late-onset muscular dystrophy characterised by slowly progressive ptosis, dysphagia, and proximal limb muscle weakness. The short expansion of a GCG or GCA trinucleotide repeat (from GCN_10_ to GCN_11–17_) encoding a polyalanine tract at the N terminus of the polyadenylate (polyA) binding protein nuclear 1 gene (*PABPN1,* also known as *PABP2*) is a common cause of OPMD. OPMD with *PABPN1* mutation is distributed worldwide, and some cases have also been reported in East Asians [1–5]. Muscle MRI studies have revealed that a pattern of tongue, hamstring, and calf muscle involvement could be considered characteristic of OPMD [6, 7]. In 2005, Robinson and colleagues were the first to report that OPMD was caused by a c.35G > C point mutation in the *PABPN1* gene, which resulted in p.Gly12Ala amino acid substitution and an increase in the number of contiguous polyalanine codons mimicking the *PABPN1* triplet repeat effect [8]. Although they reported two further cases with the point mutation in the UK [9], no cases with the point mutation were reported from other countries. We herein report a Japanese case of OPMD due to *PABPN1* c.35G > C point mutation. This is the first report of a case with genetically confirmed OPMD with a rare point mutation from an Asian country.

## Case presentation

**Fig. 1:**
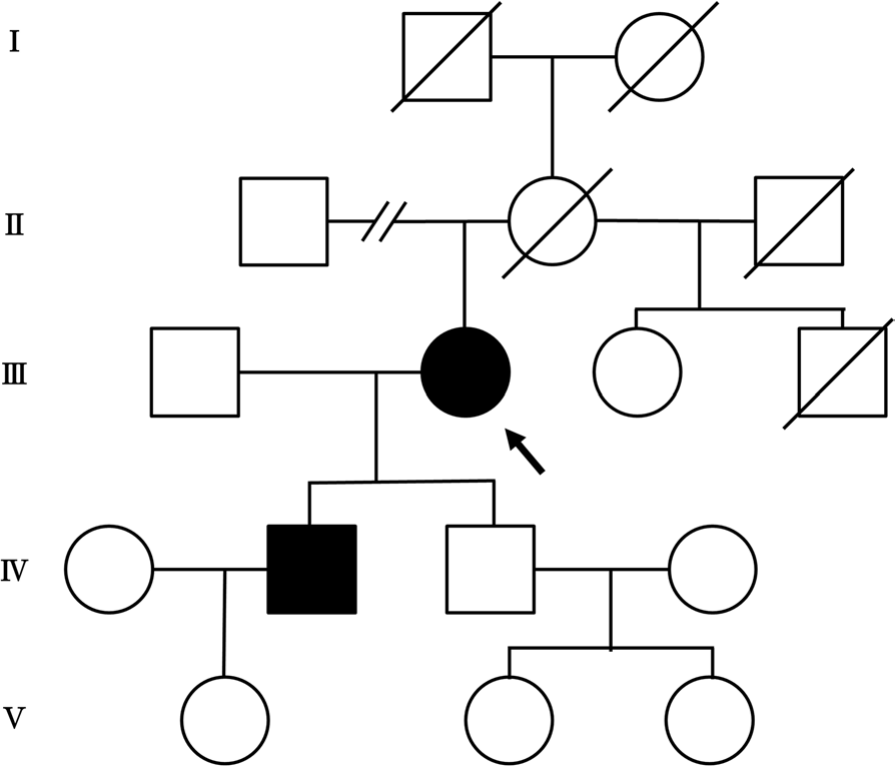
Pedigree of a Japanese family with oculopharyngeal muscular dystrophy with c.35G > C point mutation in the *PABPN1* gene. Filled symbols indicate symptomatic at present

A 78-year-old Japanese female was admitted with repeated aspiration pneumonia. She presented with bilateral ptosis and dysphasia that had gradually progressed since she was 62 years of age. She had a history of levator muscle resection at 66 years of age; however, her bilateral ptosis recurred five years after the operation. She underwent gastrostomy after admission since her swallowing function was significantly impaired. Her son had the same symptoms (ptosis and dysphasia), but the clinical history of her parents was not available (Fig. 1).

A physical examination of the patient revealed muscle weakness of the neck and limb muscles with muscle atrophy. Her muscle strength based on the Medical Research Council (MRC) grading system showed symmetrical proximal-dominant upper and lower limb and neck muscle weakness of the neck extensor (MRC 4), neck flexor (MRC 2), deltoid (MRC 4), pectoralis major (MRC 3), latissimus dorsi (MRC 4), iliopsoas (MRC 3), quadriceps (MRC 4), gluteus maximus (MRC 2), and biceps femoris (MRC 2) muscles. She had no weakness in the tibialis anterior and gastrocnemius muscles. Although she could walk independently, she required assistance to climb stairs and could not run. She had a nasal voice and difficulty in swallowing and speaking in the absence of tongue muscle atrophy.

**Fig. 2:**
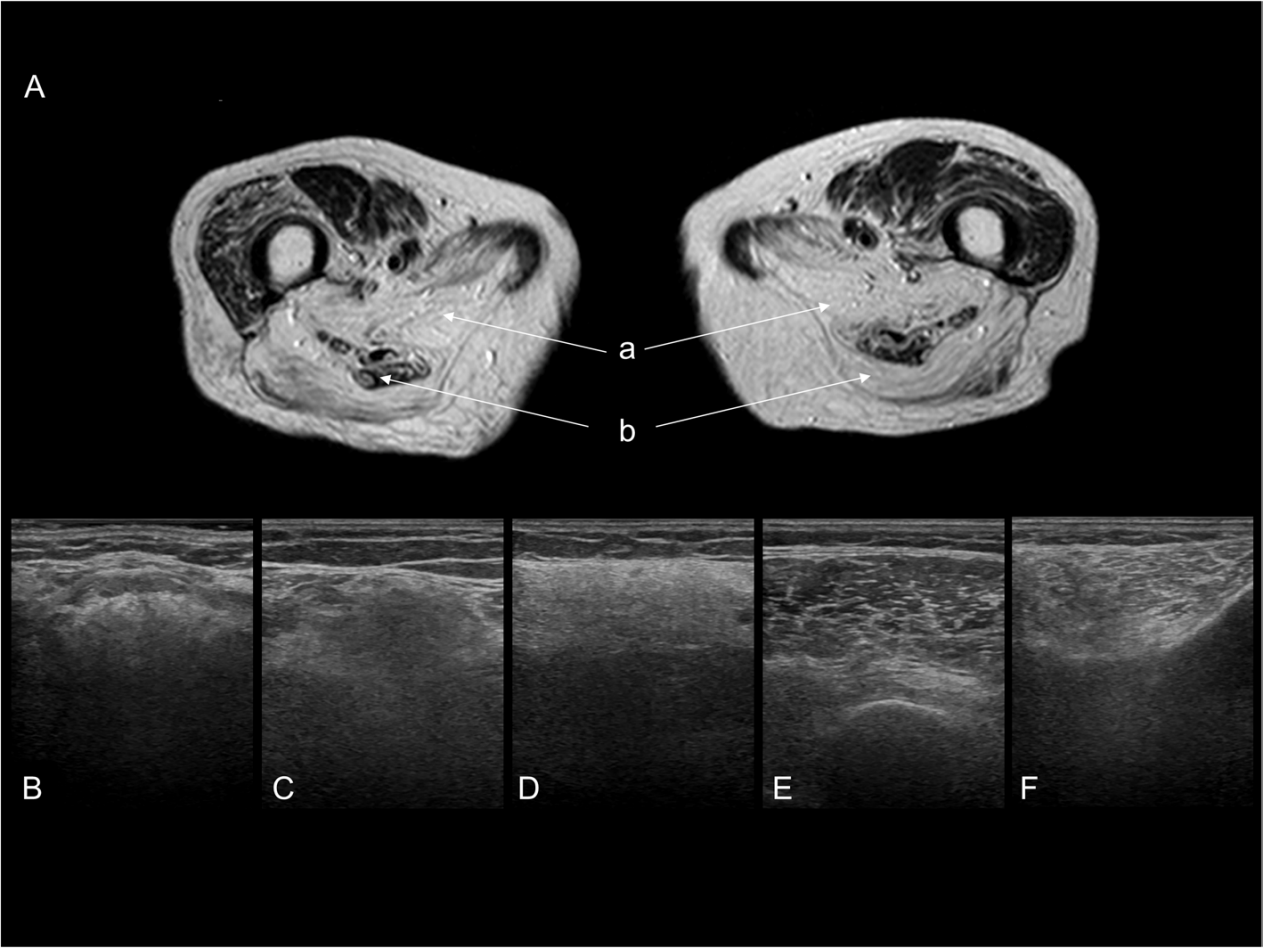
Axial T2-weighted muscle MRI at the thigh level (**A**) and muscle ultrasound images (**B-F**). On MRI, muscles in the posterior compartment (biceps longus head (**a**) and adductor magnus (**b**) muscles) showed a high signal intensity, whereas muscles in the anterior compartment were preserved (**A**). Muscle ultrasound also showed a posterior dominant-involvement pattern in lower limb muscles in addition to tongue muscle involvement. The tongue (**B**), biceps femoris (**C**), and gastrocnemius (**D**) muscles had a diffusely hyperechoic pattern, whereas normal muscle structures were relatively preserved in the vastus lateralis (**E**) and tibialis anterior (**F**) muscles

The results of electrocardiography revealed no abnormalities. The creatinine kinase level was not elevated (45 U/L). Anti-AchR and anti-Musk antibodies were negative. Arterial blood gas analysis revealed a mild increase in CO_2_ (45.9 mmHg). Muscle imaging (MRI and muscle ultrasound) showed impairment of the tongue, proximal muscles of the upper limbs, and flexor muscles of the lower limbs, being consistent with the clinical presentation (Fig. 2). Repetitive nerve stimulation testing yielded normal results. Needle-electromyography (needle-EMG) identified low amplitude and multiphasic motor unit potentials without active denervation potentials in her tongue and orbicularis oculi muscles, which indicated a chronic myopathic change.

Based on slowly progressive bilateral ptosis and dysphagia, the symmetrical proximal-dominant muscle involvement without myocardial disorder, the patient’s family history that suggested an autosomal dominant pattern of inheritance, and the myopathic pattern on needle-EMG, we initially suspected OPMD. This was confirmed by direct sequencing of *PABPN1*, which revealed a heterozygous c. 35G > C (p. Gly12Ala) point mutation. We could not perform gene analysis of her son.

## Discussion and conclusions

This is the first report of a Japanese case of OPMD due to *PABPN1* c.35G > C; p.Gly12Ala mutation, which is rare and has only been reported from the UK [8, 9]. In most cases of OPMD, the underlying cause is abnormal short (GCN)11–17/polyalanine expansions in the *PABPN1* (previously abbreviated as *PABP2*) gene on chromosome 14q11 (the normal allele is (GCG)6(GCA)3(GCG)1 encoding 10 alanines) [10]. Our patient was negative for the repeat expansion mutation. Instead, a point mutation in *PABPN1* adjacent to the 3’ end of the normal polyalanine codon repeat sequence was identified. The G → C substitution changes a glycine GGG codon, located immediately 3’ to the normal 10 alanine codon repeat sequence, to an alanine GCG codon. This change causes an increase in the number of contiguous polyalanine codons from 10 to 13 alanines without gene expansion ((GCG)6(GCA)3(GCG)2(GCT)1(GCG)1), which has the same effect as the common triplet repeat expansion mutation in OPMD [8]. Our case may suggest that the *PABPN1* point mutation causing OPMD is not only a founder effect mutation of European ancestry but has also become distributed worldwide independently.

Up to the present, three OPMD cases with the point mutation have been reported from the UK [8, 9]. Clinical features of our Japanese case were similar to those of these three previous cases. The age at onset in all cases, including our patient, was in their 60 s, and ptosis and dysphagia were the main symptoms. Limb muscle weakness was present in our patient. Among the three previous cases, two showed no limb muscle weakness at the time of diagnosis when they were 65 and 67 years old, respectively. The other previous case with limb muscle weakness was assessed and diagnosed at the age of 75. Our patient was diagnosed at the age of 78 years old, and neck and limb muscle weakness was already evident on examination. Based on these facts, trunk and limb muscle weakness may develop with advanced disease, specifically in OPMD patients in their 70 s with the point mutation. In OPMD patients with typical short polyalanine expansions, a previous study identified a negative correlation between the mean age at diagnosis and number of repeats, although phenotype-genotype correlations were not confirmed [11]. In the previous study, the mean age at diagnosis was 64 years old when the patient had 13 GCN repeats, whereas the mean age at onset of the four cases with point mutation, including our case, was 71 years old. This difference in onset age between OPMD patients with 13 repeats and point mutation raises the hypothesis that disease progression in OPMD patients with point mutation is slower than in OPMD patients with the 13 GCN repeats, although clinical manifestations of OPMD patients with point mutation are similar to those of typical OPMD cases with a short expansion of GCN [3, 5, 12]. More case data need to be accumulated to elucidate the clinical features of OPMD with point mutation because the number of patients is limited.

When we examine patients with elderly-onset dysphagia and limb weakness, the diagnoses of myasthenia gravis, amyotrophic lateral sclerosis, and inclusion body myositis are more common than the diagnosis of OPMD. In our case, muscle MRI and an extensive ultrasound examination led us to suspect OPMD. Regarding muscle selectivity, previous studies revealed that tongue, hamstring, and calf muscles were mainly affected in patients with OPMD with a short expansion of GCN, which contrasts with the quadriceps-dominant pattern seen in inclusion body myositis [6, 7]. We confirmed that OPMD patients with point mutation also exhibited the same pattern of muscle impairment as those with repeat expansion.

In conclusion, this is the first reported Japanese case of OPMD due to *PABPN1* point mutation, in which clinical features were similar to those of previous cases reported from the UK. Our case suggests that *PABPN1* point mutation should be considered as a cause of OPMD in Asian populations in addition to GCN repeat expansion.

## Data Availability

The datasets used and/or analysed during the current study available from the corresponding author on reasonable request.

## Abbreviations

OPMD: Oculopharyngeal muscular dystrophy
MRC: Medical Research Council
EMG: Electromyography
